# The acute effect of match-play on hip range of motion and isometric strength in elite tennis players

**DOI:** 10.7717/peerj.7940

**Published:** 2019-11-11

**Authors:** Victor Moreno-Pérez, Fabio Yuzo Nakamura, Violeta Sánchez-Migallón, Raul Domínguez, Valentín Emilio Fernández-Elías, Jaime Fernández-Fernández, Alberto Pérez-López, Alvaro López-Samanes

**Affiliations:** 1 Center for Translational Research in Physiotherapy, Department of Pathology and Surgery. Universidad Miguel Hernández, Elche, San Juan, Spain; 2 Associate Graduate Program in Physical Education UPE/UFPB, Joao Pessoa University, Joao Pessoa, Brazil; 3 The College of Healthcare Sciences, James Cook University, Townsville, Australia; 4 School of Physiotherapy, School of Health Sciences, Universidad Francisco de Vitoria, Pozuelo de Alarcón, Madrid, Spain; 5 College of Health Sciences, Isabel I University, Burgos, Spain; 6 Faculty of Sport Sciences, Universidad Europea de Madrid, Villaviciosa de Odón, Madrid, Spain; 7 Department of Physical Activity and Sports Sciences, Universidad del León, Spain, University of Leon, Leon, Spain; 8 Department of Biomedical Sciences (Area of Sport and Physical Education), Faculty of Medicine and Health Sciences, University of Alcala, Madrid, Spain

**Keywords:** Hip, njury prevention, ange of motion, ennis, trength, GPS

## Abstract

**Background:**

Groin injuries are some of the most common injuries tennis players suffer. Several factors (e.g., post-match decrease in hip adductor (ADD) strength) have been proposed as possible mechanisms for increasing the incidence of this type of injury. However, the risk factors of developing groin injuries after a tennis match have not yet been delineated.

**Objective:**

The aim of this study was to determine the effect of tennis match-play on isometric ADD and abductor (ABD) strength and passive hip range of motion (ROM).

**Methods:**

Twenty-six male tennis players (20.30 ± 4.98 years) took part in this study. Participants completed an evaluation of strength and flexibility hip measurements before and after a simulated tennis match. Dominant and non-dominant passive hip ROM, ADD and ABD isometric strength, and the ADD/ABD strength ratio were measured before and immediately post-match. A global positioning system (GPS) and a session rating of perceived exertion (RPE) were used to assess the locomotive demands and internal match load.

**Results:**

Isometric dominant ADD strength (17.8%, *p* ≤ 0.01) and ADD/ABD strength ratio (11.6%, *p* = 0.04) were lower post-match compared to the pre-match values. No between-limbs differences were observed for isometric ADD strength, ABD strength, and passive hip ROM tests. RPE showed an expected increase between pre- vs. post-match (pre- vs. post-warming-up, 3.42 ± 2.08 vs. 5.62 ± 2.29, *p* < 0.01). In addition, a significant relationship between ADD strength and the volume of tennis practice per week was found, stablishing that tennis players with lower volume of training per week suffered a reduction in ADD strength in their dominant limb after match-play (*r* = 0.420, *p* = 0.04).

**Conclusion:**

The assessment of ADD strength and the ADD/ABD strength ratio in the dominant limb may be considered a post-match tool that can be used to identify players who require rest and additional recovery strategies before competing again.

## Introduction

Tennis is an intermittent sport characterized by high-intensity efforts interspersed with periods of low-intensity activity (e.g., active recovery between points and rest between changeover breaks) over a variable period (i.e., 1–5 h) (López-Samanes et al., 2017). During a tennis match, the players cover around 1,500–3,100 m (Gallo-Salazar et al., 2015; Pereira et al., 2016) and report ratings of perceived exertion (RPE) of ~5.5 units after matches (Murphy et al., 2016). Due to the variability of match length during a competition, physical performance during prolonged matches may drop due to the appearance of neuromuscular fatigue signs (Girard et al., 2008) manifested as a decrease in maximal voluntary contraction and leg stiffness values (Girard et al., 2006). Importantly, neuromuscular fatigue during competition has also been associated with an increased injury risk to the upper limbs due to several factors, including scapular upward rotation deficits (Rich et al., 2016) or reductions in strength values in the shoulders after tennis matches (Moreno-Pérez et al., 2019). However, no reductions in hip strength have been found in young tennis players after two matches (Gallo-Salazar et al., 2017); and to our knowledge, no previous studies have focused on elite tennis players.

Groin injuries are common injuries in elite junior tennis players (Hutchinson et al., 1995; Pluim et al., 2016) with a reported incidence of 2.84 groin injuries per 1,000 playing hours in tennis matches (Sell et al., 2013), accounting for 25% of all acute injuries elite junior tennis players suffer (Pluim et al., 2016). Groin injuries are commonly produced during sudden changes of direction while running at high speeds, specifically when attempting to stop lateral movement by sliding or posting the lead foot (Kibler & Safran, 2005); this has a significant and detrimental impact on tennis players’ performance.

To implement preventive strategies, the identification of risk factors associated with groin injury occurrence is paramount. Previous studies of other sports (e.g., tennis, soccer, ice hockey, rugby or Australian Football), have related several extrinsic and intrinsic risk factors to an increased likelihood of developing groin injuries (Moreno-Pérez et al., 2017; Ryan, DeBurca & Mc Creesh, 2014; Sedaghati et al., 2013; Tyler et al., 2001). These include the level of competition or experience (Tyler et al., 2001), decreased range of hip abduction (ABD) and rotation (Arnason et al., 2004; Ibrahim, Murrell & Knapman, 2007), isometric adductor (ADD), muscle weakness and lower ADD/ABD strength ratios (Arnason et al., 2004; Engebretsen et al., 2010; Moreno-Pérez et al., 2017; Ryan, DeBurca & Mc Creesh, 2014; Tyler et al., 2001).

Moreover, Ibrahim, Murrell & Knapman (2007) and Arnason et al. (2004) found that limited hip abduction and the total rotation range of motion (ROM) are associated with an increased risk of injury in football players. In ice hockey, players with an ADD/ABD strength ratio of less than 80% are 17 times more likely to sustain an ADD injury (Tyler et al., 2001). However, other studies did not support the relevance of this findings (Emery & Meeuwisse, 2001; Engebretsen et al., 2010). These differences may be due to the methodology used and the time of season when hip musculoskeletal characteristics were examined. The in-season cumulative load effects of training and matches seem to be decisive factors regarding athletes’ musculoskeletal parameters (Wollin, Thorborg & Pizzari, 2017). Crow et al. (2010) reinforced this notion by observing a reduction of hip ADD isometric strength preceding groin pain and injury. This suggests that a groin injury may be detected before the onset of symptoms through regular screening of ADD strength

Despite the relevance of cumulative fatigue on the hip musculoskeletal characteristics of athletes, the acute effect of a single fatiguing match on hip injury risk factors has not yet been examined. Fatigue might be an important mechanism underlying injury, since after a single football match, exercise-induced fatigue increased the rate of groin injury (Hawkins et al., 2001; Woods et al., 2004), which is typically associated with a decrease in running performance during a match (Hawkins et al., 2001; Woods et al., 2004). Only one study has analyzed the acute effects of competitive tennis match-play on ADD strength but found no significant effect (Gallo-Salazar et al., 2017). However, researchers have demonstrated that tennis match-play may affect the players’ ROM (Gallo-Salazar et al., 2017; Martin et al., 2016; Moore-Reed et al., 2016). Martin et al. (2016) analyzed the rotational shoulder ROM in young tennis players in response to a 3-h tennis match, and they found a significant decrease in passive shoulder internal rotation and total ROM after the match. These adaptations in shoulder ROM increase shoulder injury risk and negatively influence players’ serve performance during tennis match-play (Martin et al., 2016). Nevertheless, scarce evidence exists regarding hip ROM response after playing a single match in tennis players.

The first aim of this study was to examine the acute effect of competitive tennis match-play demands on clinical groin injury risk measures, such as bilateral passive hip ROM, isometric ADD and ABD strength, and the ADD/ABD isometric strength ratio, by comparing results prior to and immediately post-match. The secondary aim was to evaluate the relationship between locomotive match-play demands and the players’ characteristics (age, years of tennis experience and duration of tennis practice per week) with possible changes in ROM and hip strength.

## Methods

### Subjects

Twenty-six male tennis players (age: 20.38 ± 4.38 years; height: 1.81 ± 0.08 m; body mass: 72.02 ± 10.17 kg) volunteered to participate in the study. All the participants had an Association of Tennis Professionals ranking, International Tennis Federation ranking, or senior national ranking within the 300 best Spanish senior tennis players. Twenty-three (88.4%) players were right handed, and three (11.5%) were left handed. Players were training 25.46 ± 12.46 h/week, and they had an average of 12.04 ± 5.43 years of tennis playing experience. The inclusion criteria were (a) being healthy and actively competing at the time of the study, (b) presenting with no recent injury or surgery and not having taken any type of medication for to treat pain or musculoskeletal injuries at the time of the study, and (c) having an absence of delayed onset muscle soreness during the testing session.

Before the start of this investigation, all players were fully informed about the testing, and the written informed consent was obtained. The procedure was approved by the Institutional Ethics Review Committee (Francisco de Vitoria University 45/2018) and conformed to the World Medical Association’s code of ethics of Helsinki.

### Design

Competitive tennis matches were conducted in the facilities of different tennis academies or tennis clubs during the same month. Upon arrival, players filled out a questionnaire containing information about body mass, height, medical history, and training frequency (number of h of practice per day and week). Testing (ROM and strength) was performed in the clinical area of each tennis academy. All testing was conducted by a senior sports physiotherapist (with 19 years of experience) and another physiotherapist (2 years of experience), ensuring consistent participant positioning throughout the assessments. Based on Wollin, Thorborg & Pizzari (2017), players’ testing order and the selection of the tested leg were randomly chosen prior to the pre-match test. Pre-match testing was performed 60 min prior to match–play, and the post-match re-testing was performed immediately after the match. At the beginning of the pre-match testing, participants performed 5 min of warm-ups (jogging) and standardized dynamic warm-up exercises based on those described by Ayala et al. (2016). Subsequently, participants played the simulated tennis match against an opponent, who was matched based on playing level (i.e., according to international or national tennis ranking). All matches were played according to the rules of the International Tennis Federation on an outdoor hard-court surface (Greenset surface, Green Set WorldWide, Barcelona, Spain). The matches were played using a best-of-3-sets system with a super tiebreak during the 3rd set. The resting times allowed were 20 s between points, 90 s between changeovers, and 120 s between sets. The intensity of all matches was estimated using a global positioning system (GPS) (GPSports, Canberra, NSW, Australia) and the RPE scale. During the tennis matches, the air temperature and humidity were monitored using a portable weather station (WMR 108; Mextech, Mumbai, India). Data were averaged to obtain the mean temperature and relative humidity during the match (%). To reduce the interference of uncontrolled variables, all subjects were instructed to maintain their habitual lifestyle and normal dietary intake before and during the study.

### Methodology

The dominant limb for each of the elite tennis players was determined according to the definition given by Ellenbecker et al. (2007), who defined it as “the limb on the same side as the subject’s dominant (tennis playing) upper extremity.”

#### Hip strength testing

**Figure 1:**
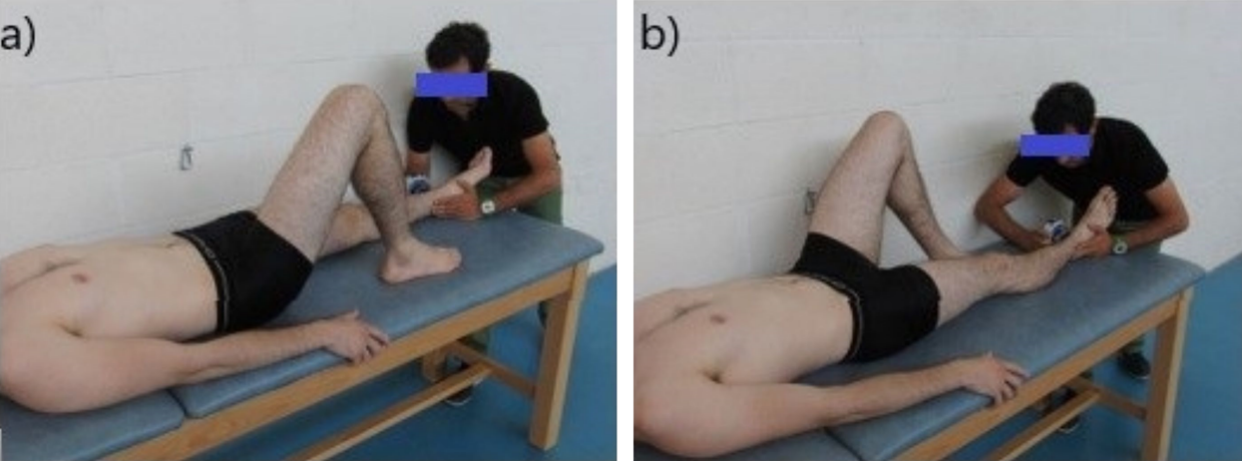
Assessment of the isometric hip strength: (A) testing for hip maximal isometric abduction strength; (B) testing for hip maximal isometric adduction strength.

To measure hip ADD and ABD isometric strength in both sides, we followed the methods previously described by Thorborg et al. (2010b) and used a portable handheld dynamometer (Nicholas Manual Muscle Tester; Lafayette Indiana Instruments, Lafayette, IN, USA). The participants were placed in the supine position with the hip in a neutral pose and were told to stabilize themselves by holding onto the sides of the table. Examiner 1 applied resistance in a fixed position that was 5 cm proximal to the proximal edge of the lateral (for ABD) or medial malleolus (for ADD) (Figs. 1A and 1B, respectively). The tennis players exerted a 5-s maximum voluntary contractions against the dynamometer and repeated the exercise three times (Thorborg et al., 2010a). There was a 30-s rest period between trials. Hip abduction strength was expressed as the maximal hip abduction torque per kilogram of body weight (Nm/kg) using the external lever arm and body weight of each participant. The intra-class correlation coefficient for this test ranged from 0.83 to 0.96.

#### Hip range-of-motion testing

**Figure 2:**
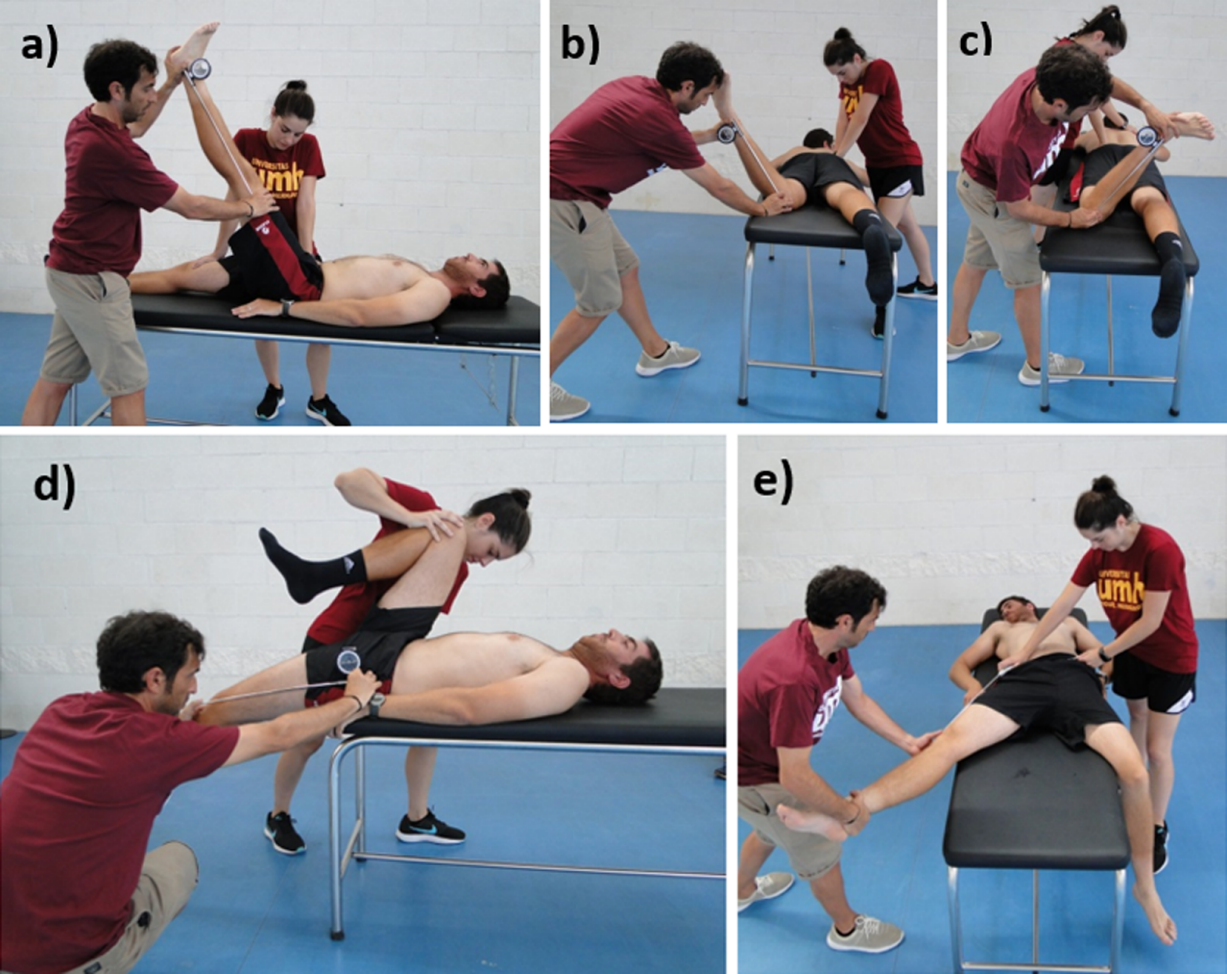
Assessment of the hip range of motion: (A) passive straight leg raise test; (B) passive hip internal rotation test; (C) passive hip external rotation test; (D) modified Thomas test; (E) hip abduction with knee extended test.

The passive straight leg raise test (Fig. 2A), internal and external rotation ROM (Figs. 2B and 2C), and hip extension (Fig. 2D) were assessed following a previously described method (Cejudo et al., 2015) and using an inclinometer (ISOMED, Portland, OR, USA) with a telescopic arm. Prior to each assessment, the inclinometer was calibrated to 0° with either the vertical or horizontal axis. The angle (following its bisector) between the longitudinal axis of the mobilized segment and the vertical or the horizontal reference lines was recorded. For the the hip abduction assessment (Fig. 2E), a flexible, adjustable long-arm goniometer Gollehon 180° was used (Moreno-Pérez et al., 2016).

The endpoint of each test was determined using at least one of the following criteria: (a) the participant had an appreciated or palpable onset of compensation movement and/or pelvic rotation or (b) the participant perceived a strong but tolerable stretch slightly prior to the occurrence of pain. The intra-class correlation coefficient ranged from 0.87 to 0.97 (Cejudo et al., 2015).

#### Internal match load

The subjective internal load of the game was obtained using the session RPE method within 30 min of match termination, which has been previously validated in different sports (Scott et al., 2013). The intensity rating (RPE value) was multiplied by the time spent playing to give a game load in arbitrary units (AUs) (Foster et al., 2001).

#### External match load

The locomotive demands of the match were quantified using GPS with data collected from all the games. The GPS units (Spi-Pro X, GPSports, Canberra, NSW, Australia) were placed between the scapulae of the players in bespoke vests. These units sampled at 15 Hz, and the accelerometers samples at 100 Hz. After each match, the data were downloaded using a specialized analysis software (TeamAMS, GPSports, Canberra, NSW, Australia). The match variables analyzed in this study provided information about the distance covered, number of accelerations, deceleration, body impacts, and body load. All the variables were obtained from the dedicated software.

### Statistical analysis

A Shapiro–Wilk test was used to assess the normal distribution of data. Data are presented as means and standard deviation. All variables were compared using a sample-dependent *t* test (pre- vs. post-match). The significance level was set at *p* < 0.05. Lankens’ formula (Lakens, 2013) for effect size, was used and the interpretation of the results was based on the following criteria: trivial (0–0.19), small (0.20–0.49), medium (0.50–0.79), and large (0.80 and greater). Performance variables, the characteristics of the players, and tennis experience were correlated (using Pearson’s *r*) with locomotive demands. All the statistical analyses were completed using the SPSS software version 25 (SPSS Inc., Chicago, IL, USA).

## Results

### Environmental conditions, match duration, and training load

The mean duration of the tennis matches was 80.3 ± 21.3 min, and a total of 16.50 ± 3.33 games were played during each match. In both testing trials (pre- and post-match), environmental conditions were similar, with a temperature of 18.4 ± 6.4 °C and a humidity of 40 ± 8%. The internal match load was approximately 451.2 AU, and RPE showed an expected increase between pre- vs. post-match (pre- vs. post-warming-up, 3.42 ± 2.08 vs. 5.62 ± 2.29, *p* < 0.01).

### Demands of tennis match-play

The total distance covered, the distance covered at different velocities, the number of acceleration/deceleration instances, the total body impacts, and the body load were recorded. The demands of tennis match-play are shown in Table 1.

**Table 1:** Demands of the match-play.

Total distance covered (m)	4085.0 ± 974.4
Distance covered at 0–6 km/h (m)	3271.2 ± 799.1
Distance covered at 6–12 km/h (m)	642.1 ± 177.7
Distance covered at 12-14 km/h (m)	149.1 ± 123.4
Distance covered at 14–18 km/h (m)	56.9 ± 44.4
Distance covered >18 km/h (m)	12.6 ± 15.5
Number of accelerations (>3m/s^2^)	22.8 ± 12.1
Sum of accelerations (m)	124.3 ± 69.0
Number of decelerations (1.2–2.4 m/s^2^)	22.4 ± 12.2
Number of decelerations (2.4–3.6 m/s^2^)	8.4 ± 5.1
Number of decelerations (>3.6 m/s^2^)	2.2 ± 2.8
Body impacts (>5g)	3033.6 ± 788.4
Body load (au)	45.6 ± 21.4

**Note:**

Abbreviations: m, meters; km/h, kilometers per hour; au, arbitrary units.

### Strength and ROM hip values

**Figure 3:**
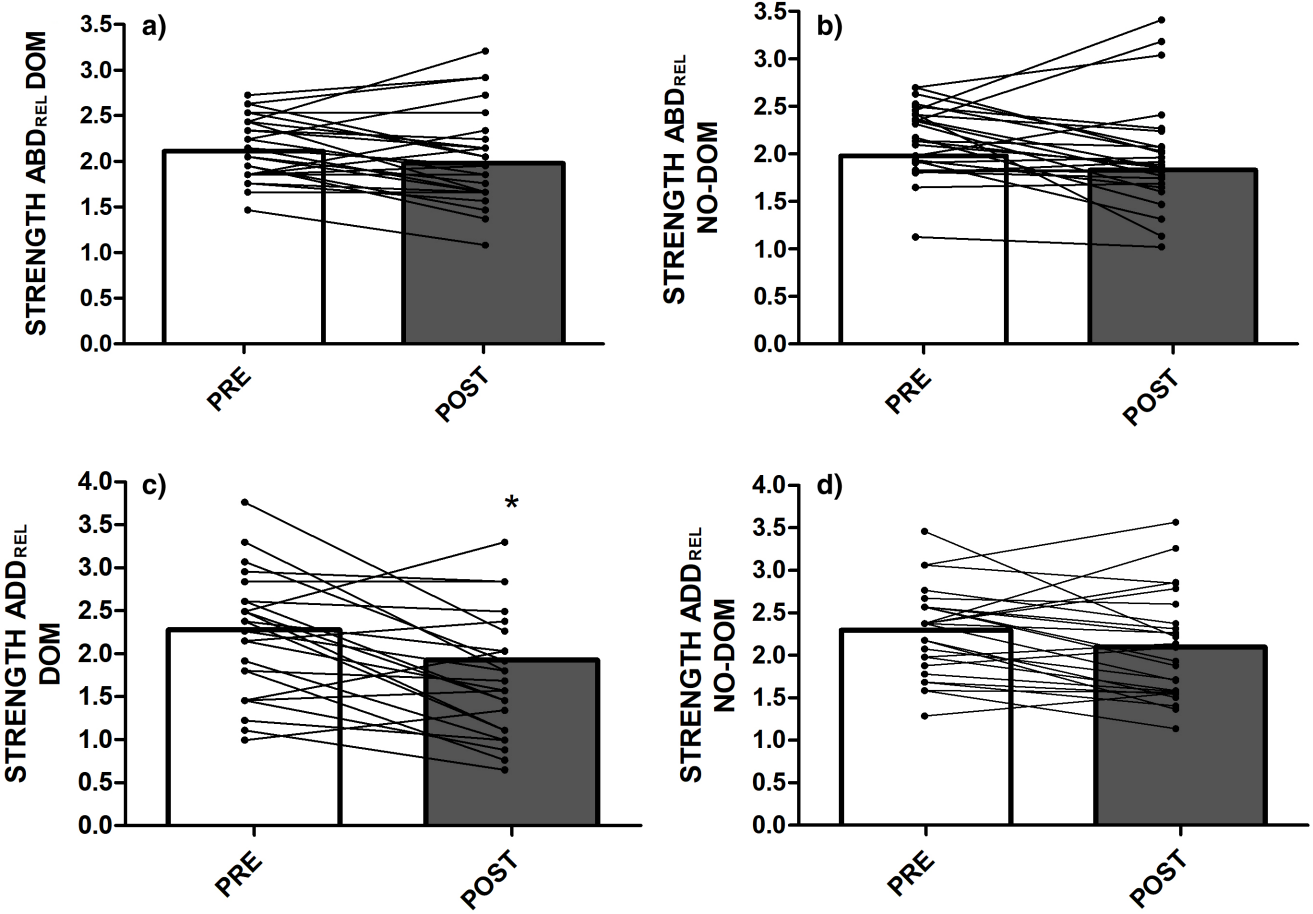
Hip isometric strength values: (A) Relative Hip abductor strength ratio in the dominant limb; (B) Relative Hip abductor strength ratio in the non-dominant limb; (C) Relative Hip adductor strength ratio in the dominant limb; (D) Relative Hip adductor strength ratio in the non-dominant limb. Abbreviations: DOM = dominant; NO-DOM = non-dominant; ADD = Adductor; ABD = Abductor; REL = relative. *Significant differences compared to the PRE values at *P* < 0.05.

**Figure 4:**
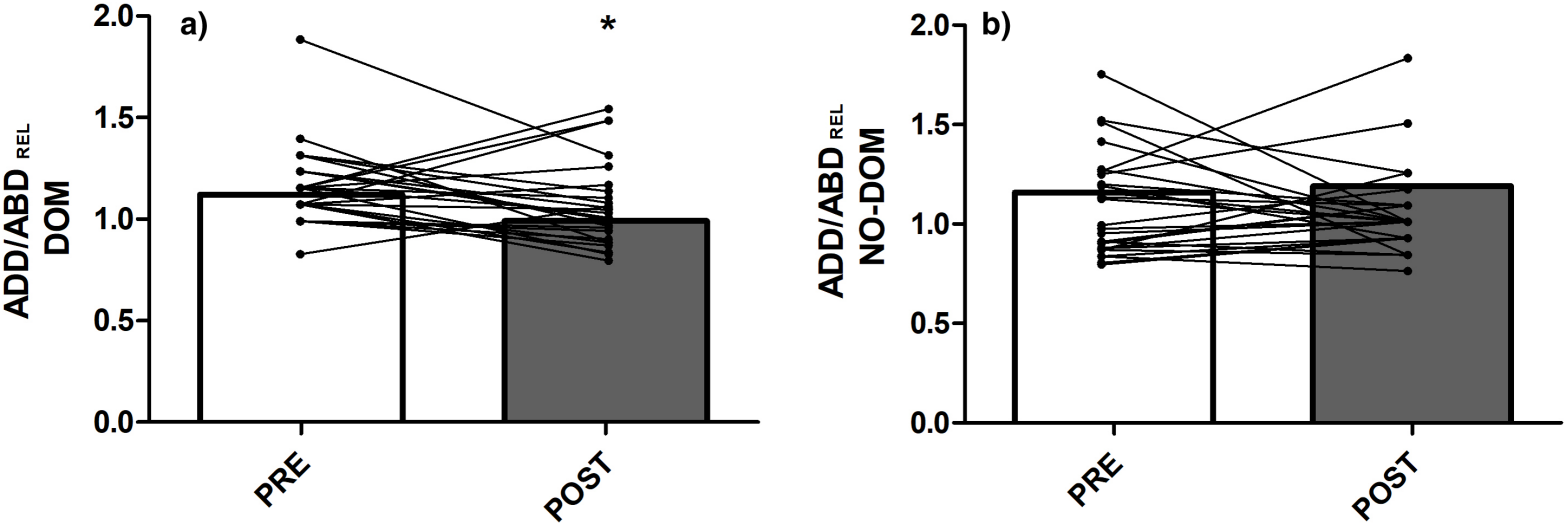
ADD–ABD strength ratios: (A) Isometric hip adduction/abduction ratio in the dominant limb; (B) Isometric hip adduction/abduction ratio in the non-dominant limb. Abbreviations: DOM = dominant; NO-DOM = non-dominant; ADD = Adductor; ABD = Abductor; REL = relative. *Significant differences compared to the PRE values at *P* < 0.05.

The mean values of bilateral passive hip ROM are presented in Table 2. The isometric ABD and ADD strength and the ADD/ABD isometric strength ratio measurements, pre- and post-match, are illustrated in Figs. 3 and 4, respectively. Adduction strength values and ADD/ABD strength ratios on the dominant limb were significantly lower (17.8% and 11.6%, respectively) post-match compared with the pre-match values (ES CI [0.284, 1.201] and [0.080, 0.938], *p* ≤ 0.01 and *p* = 0.05, respectively). However, no between-limbs differences were observed for isometric ABD strength and passive hip ROM tests in pre- and post-match moments.

**Table 2:** Hip ROM and isometric strength.

Variables	Pre-match (*N* = 26)	Post-match (*N* = 26)	*p*	ES [95% CI]
ROM IR D	36.39 ± 9.52	36.26 ± 10.76	0.946	−0.072 [−0.304, 0.329]
ROM IR ND	34.83 ± 9.88	33.32 ± 10.66	0.305	0.039 [−0.146, 0.442]
ROM ER D	42.69 ±11.78	43.30 ± 9.04	0.787	−0.022 [−0.379, 0.278]
ROM ER ND	42.67 ±13.89	46.42 ± 9.25	0.124	−0.181 [−0.59, 0.066]
ROM EX D	3.03 ± 10.69	2.75 ± 8.87	0.828	−0.04 [−0.308, 0.361]
ROM EX ND	4.75 ± 10.91	3.88 ± 8.86	0.474	0.003 [−0.251, 0,405]
ROM FL D	77.75 ± 8.66	74.15 ± 9.91	0.109	0.303 [0.101, 0.938]
ROM FL ND	75.55 ± 8.76	74.82 ± 10.03	0.717	−0.054 [−0.186, 0,348]
ROM ABD D	36.27 ± 8.18	37.07 ± 7.47	0.350	0.049 [−0.353, 0.162]
ROM ABD ND	36.48 ± 7.96	37.61 ± 7.66	0.222	−0.005 [−0.407, 0.131]
Strength ABD _REL_ D	2.10 ± 0.32	1.97 ± 0.53	0.108	0.291 [0.064, 0.708]
Strength ABD _REL_ ND	2.02 ± 0.10	1.83 ± 0.58	0.058	0.433 [0.161, 1,02]
Strength ADD _REL_ D	2.35 ± 0.54	1.93 ± 0.62	0.001	0.658 [0.366, 1,283]*
Strength ADD _REL_ ND	2.29 ± 0.49	2.10 ± 0.61	0.050	0.29 [0.056, 0.888]
Ratio_ADD/ABD _REL_ D	1.12 ± 0.24	0.99 ± 0.25	0.040	0.446 [0.143, 0,875]*
Ratio_ADD/ABD _REL_ ND	1.16 ± 0.27	1.19 ± 0.31	0.594	−0.069 [−0.472, 0.333]

**Note:**

Abbreviations: IR, Internal rotation; ER, External rotation; EX, Extension; FL, Flexion; D, Dominant; ND, Non-dominant; ABD, Abductors; ADD, Adductors; REL, relative; ES, Effect size mean [95% confidence limits] *statistically significant between pre- and post-match difference (*p* < 0.05).

### Correlations

Pearson’s correlation coefficients showed a significant relationship between ADD strength and the volume of tennis practice per week (*r* = 0.420, *p* = 0.04) in the dominant limb. However, the remaining locomotive match-play demands (meters per minute, total distance, number of accelerations, number of decelerations, number of impacts, and body load), players’ characteristics (age and years of tennis experience), and changes in ADD strength did not result in significant correlations (*p* = 0.05–0.86, *r* = −0.365 to 0.347). All correlations were performed by normalizing ADD and ABD strength data to body mass (N/Kg).

## Discussion

Groin injuries are common in tennis (Pluim et al., 2016). Researchers have suggested a decrease in hip ADD strength and hip ROM after match-play occurs in several sports (Ryan, DeBurca & Mc Creesh, 2014; Wollin, Thorborg & Pizzari, 2017), and this may be an important risk factor for developing groin injuries. To the best of our knowledge, very few studies have evaluated the transient changes of hip ROM and adduction and abduction strength after match-play across different sports (Paul et al., 2014; Roe et al., 2016), and only one did so in young tennis players (Gallo-Salazar et al., 2017).

Lower limb muscle strength/power production is critical to executing explosive actions in tennis (e.g., acceleration, changes in direction) (Girard & Millet, 2009). In this study, a significant reduction in dominant ADD muscle strength (17.8%, ES = 0.74) was observed after a single tennis match. A study of 14 rugby players showed only trivial ADD muscle In the study by, a cohort of 14 rugby players showed trivial ADD muscle weakness (−1.3 ± 2.5%) after match-play (Roe et al., 2016). The greater reduction in ADD strength in this study may be explained by the demands of tennis matches as players are required to perform multiple short high-intensity movements (e.g., acceleration, deceleration, and rapid changes in direction), which imposes an elevated concentric and eccentric load on the ADD muscles. Another study by Gallo-Salazar et al. (2017) found a significant improvement in isometric hip ADD strength in the dominant and non-dominant sides (3.3% and 6.7%, respectively) in young tennis players after two matches. This different finding could be related to the characteristics of the samples used in the studies (i.e., young vs. senior tennis players). For instance, our senior tennis players covered around 22–54% more distance during the match than in other studies involving tennis players (Gallo-Salazar et al., 2017; Pereira et al., 2017), although the RPE values found after match (i.e., 5.62) agree with previous reports (Murphy et al., 2016). Therefore, the lack of agreement between studies may be due to differences in recording methods and/or the demands of the participants’ match-play. Furthermore, in Gallo-Salazar et al. (2017), the tennis players did not perform a warm-up prior to the testing sessions. In this study, all participants performed a dynamic-stretch warm-up according to the procedures adopted by Ayala et al. (2016). Another possible explanation for the noted differences in post-match ADD strength could result from the different demands of match of the participant´s match play (e.g., intensity, duration, frequency, etc). The study by Gallo-Salazar et al. (2017) did not quantify the demands of the match-play in twelve young players with an average age of 14.4 ± 0.9 years, while our study found average of distances covered of 4085.0 ± 974.4 m in 26 tennis players with an average age of 20.4 ± 4.4 years. Future research involving tennis players needs to be carried out to elucidate the effects of the different load demands imposed during matches on the ADD muscle strength in different samples of tennis players and on different court surfaces.

Several authors suggest that ADD/ABD strength ratio deficits of 10–16.4% may increase the risk of groin injuries in tennis players (Moreno-Pérez et al., 2017) and 20% in hockey players (Tyler et al., 2001). In this study, the ADD/ABD strength ratio was reduced in the dominant limb (11.6%) in response to a tennis match. It can be speculated that the lower post-match ADD strength obtained in the dominant limb may indicate a greater imbalance between the ADD/ABD muscle strength ratio after match-play compared with pre-exercise levels. Future research in tennis players is needed to elucidate the effects of different training and competition demands on ADD strength as a risk factor for groin injuries.

No significant pre-to-post-match differences in hip ROM were observed in this study. Previous studies have found reduced rotational shoulder ROM in professional (Moreno-Pérez et al., 2019), young (Gallo-Salazar et al., 2017) and elite tennis players (Martin et al., 2016) following tennis match-play. The differences post-match between hip and shoulder ROM could be due to the different movement patterns and joint activities required between the upper and lower body (Moreno-Pérez et al., 2016). The findings of this study were similar to those of previous studies using similar methodology (Charlton et al., 2018; Wollin, Thorborg & Pizzari, 2017) conducted with athletes practicing other sports that entail repeated multidirectional and cutting movements. However, other studies using different methodology have reported a significant relationship between hip ROM (i.e., a hip ABD test) and match-play activity in football players (Paul et al., 2014). Paul et al. (2014) used the bent-knee fall-out test to measure passive hip ROM, a measure combining passive hip abduction, external rotation and extension motion rather than a specific and isolated measure of hip abduction. In this study, we used more specific testing maneuvers to measure hip abduction ROM (these involved isolated hip movement), minimizing the possible compensatory hip movements with the aid of an assistant physical therapist. Training and playing exposure were also different between the two cohorts. Paul et al. (2014) reporting an average of 8 h/week of training, while the uninjured group in the current investigation was exposed to 25.46 ± 12.46 h/week of training making the players investigated in this study at higher risk of injury in terms of training exposure.

Another interesting finding of this study included the positive relationship between ADD strength in the dominant limb and the volume of tennis practice per week. According to this association, the hip ADD strength of the dominant limb could be influenced by the tennis workload during the week. Previous literature indicated that inadequate training loads (both under- and overload) would increase injury risk and reduce fitness (Gabbett, 2016). Despite the intriguing training load–ADD strength relationship reported in our study, future experimental studies are required to confirm the association between these two variables.

Finally, the changes in hip ROM and ADD muscle strength were only evaluated at the end of the match-play in this study, and future studies should examine these variables several times during the first 48 h after match-play to track the recovery kinetics prior to the following match or training session.

## Conclusion

Significantly weaker isometric hip ADD strength and smaller ADD/ABD strength ratios in the dominant limb of elite tennis players are evident after match play. However, no differences were found in the isometric ADD and ABD strength in the non-dominant limb, or in bilateral passive hip ROM between the pre- and post-match examinations. Ideally ADD strength should be restored before playing again.

## Supplemental Information

10.7717/peerj.7940/supp-1 Supplemental Information 1 Raw data.

## Additional Information and Declarations

### Data Availability

The following information was supplied regarding data availability: The raw measurements are available in the Supplemental File.
